# Nine keys for successful interprofessional collaboration Based on observing facilitators and barriers during different types of treatment meetings: A qualitative study

**DOI:** 10.1371/journal.pone.0350554

**Published:** 2026-07-01

**Authors:** Simon T. de Gans, Renee C. M. A. Raijmann, Meike F. Huijbers, Natasja Looman, Marjolein van de Pol, Babette C. van der Zwaard, Huiberdina L. Koek, Marielle H. Emmelot-Vonk, Carolina J. P. W. Keijsers

**Affiliations:** 1 Department of Geriatrics Jeroen Bosch Hospital, GZ ‘s-Hertogenbosch, The Netherlands; 2 Department of Geriatrics University Medical Centre Utrecht, Utrecht, The Netherlands; 3 Department of Primary and Community Care University Medical Centre Nijmegen, Nijmegen, The Netherlands; 4 Jeroen Bosch Academy Research, Jeroen Bosch Hospital, GZ ‘s-Hertogenbosch, The Netherlands; Faculty of Health Sciences - Universidade da Beira Interior, PORTUGAL

## Abstract

**Introduction:**

The increasing prevalence of multimorbidity requires effective collaboration between health professionals. Both interprofessional (IP) and multidisciplinary (MD) collaboration can be used for this purpose. IP collaboration refers to professionals working together toward a common patient-centred goal. In contrast, MD collaboration involves health professionals advocating for their own specialty, which may result in different disease-centred goals for the same patient. This study aims to evaluate and compare these two types of collaboration and develop recommendations for successful collaboration for patients with multimorbidity.

**Methods:**

This qualitative study employed a rapid ethnographic non-participatory approach. We observed IP and MD treatment meetings using video and audio recordings to identify factors that might influence collaboration between health professionals. Data were analysed iteratively by multiple researchers using a thematic and conventional content analysis.

**Results:**

There were clear differences between the observed meetings. The IP meetings had a roundtable setting, designated chair, discussions around a common patient-centred goal, active participants, and a relaxed atmosphere that facilitated interprofessional learning. Conversely, the MD meetings had a theatre design, disease-centred discussion, more passive participants, and a less relaxed atmosphere with fewer opportunities for learning. Five participants attended both settings. These participants were more actively engaged and contributed more to interprofessional learning in the IP meetings than in the MD meetings.

**Conclusion:**

This study showed multiple factors that may influence collaboration and participants’ behaviour, particularly regarding active participation, learning, and patient-centred care. These factors were translated into nine keys for optimizing collaboration, which could support improvement in collaborative practice.

## Background

Optimal collaboration among healthcare professionals is of the utmost importance for delivering the highest quality of care. Nowadays, effective collaboration is even more urgent due to the increasing life expectancy and the prevalence of multimorbidity [1,2]. Patients with multimorbidity are often treated by multiple different medical specialists. As the number of involved healthcare professionals increases, so does the complexity of coordinating care, putting these patients at risk of receiving fragmented care [3,4]. Care fragmentation can lead to poorer quality of care, avoidable hospitalizations, and higher costs [5]. In contrast, effective collaboration can ensure the coherence and quality of care and thus prevent care fragmentation [6]. To promote effective collaboration, the WHO has recommended interprofessional collaboration in its 2010 framework. [7].

**Fig 1:**
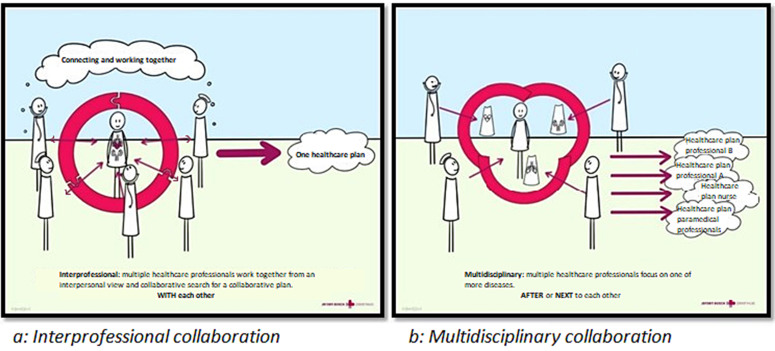
Differences between interprofessional and multidisciplinary forms of collaboration.

Although the terminology of different types of collaboration is not used consistently in the literature and may vary between countries [8], two commonly recognised approaches are interprofessional and multidisciplinary collaboration. Fig 1 illustrates the similarities and differences.

The term ‘Interprofessional collaboration’ is used internationally to describe collaboration among different healthcare professionals who contribute knowledge and skills and work together as an effective team to provide patient-centred care [7,9], see Fig 1a. Interprofessional care results in a single, patient-centred treatment plan in which all perspectives are considered and balanced in terms of the individual, not just the organ or disease. Another commonly used term is ‘intraprofessional collaboration’, which can be seen as a subset of interprofessional collaboration [10]. Intraprofessional collaboration refers to individuals from different disciplines within a single profession working together (e.g., different doctors such as cardiologists and pulmonologists), whereas interprofessional collaboration refers to individuals from different professions working together (e.g., nurses and doctors) [11]. This study will only use the term interprofessional collaboration, as both interprofessional and intraprofessional collaboration could be used interchangeably.

In contrast, the term multidisciplinary collaboration refers to a widely used approach in which professionals from different disciplines work alongside each other, focusing on their own specific specialty and/or organ of interest, and may not consider the entirety of the patient’s wellbeing [7,9]. As a result, unlike interprofessional collaboration, multidisciplinary collaboration does not always result in a unified treatment plan, see Fig 1b [9,12]. Instead, it may result in several different organ-specific plans, with the combined recommendations not always leading to the best outcome for the patient. For example, the nephrologist may recommend increased fluid intake to support renal function, whereas the cardiologist may recommend fluid restriction to optimise the cardiac condition in the same patient.

Many studies have investigated factors influencing the efficacy of interprofessional and multidisciplinary collaboration, identifying both facilitators and barriers. Factors that facilitate collaboration are well-defined professional rolls, a collaborative goal, trust, mutual respect, and a safe learning environment [13–19]. Conversely, potential barriers to effective collaboration include power dynamics, hierarchy, disrespect, current or past conflict, poor communication, and distractions [13–16]. Previous research has shown that successful collaborations can lead to improvements in patient care, such as a reduction in the length of hospital stay [16,20].

While interprofessional and multidisciplinary collaboration have been studied individually, there is limited literature directly comparing the facilitators and barriers to collaboration in interprofessional versus multidisciplinary meetings. Therefore, the aim of this study is to identify and compare the facilitators and barriers to collaboration during interprofessional and multidisciplinary meetings. Based on these factors, this study aims to develop recommendations that could promote future strategies for collaboration between health professionals.

## Methods

### Design

We carried out a qualitative study using a non-participatory rapid ethnographic research approach to examine and compare two meeting types: interprofessional (IP) and multidisciplinary (MD) meetings. Fig 1 shows the definition of these types of meetings. We observed the IP and MD meetings using video and audio recordings, and described factors that might influence collaboration between healthcare professionals in clinical practice. By adopting a social constructivist research paradigm, we investigated the relationships and social interactions between participants by observing their interactions and individual behaviours [21]. This approach enabled researchers to gain insights into the social phenomena and socio-cultural dimensions of different forms of collaboration during treatment meetings [22]. Ethnographic methods offer an efficient means of gathering data within a limited timeframe using triangulation [23].

The SRQR (Standards for Reporting Qualitative Research) guideline was used in the design and reporting of this study.

### Setting and study population

This study was conducted at the Jeroen Bosch Hospital, a large Dutch teaching hospital between 15 June 2023 and 1 August 2023. We identified one IP meeting and one MD meeting according to the definitions described in the introduction section. The selected IP meeting was the Intensive Collaboration Ward (ICW) treatment meeting, and the MD meeting was the endocarditis treatment meeting. When selecting these meetings we searched for two meetings that had similar case complexity and participants, so that any differences observed in behaviour or collaboration might be explained by the different meeting format. The ICW meeting and the endocarditis meeting had similar case complexity and the team consisted of similar participants. Some patients were discussed at both meetings. And the cardiologists and internal medicine specialists were present in both meeting types., It should be noted that treatment meetings for similar categories of patients in other hospitals may be either IP or MD, depending on their specific characteristics.

The ICW is a hospital ward where older patients with multimorbidity are admitted who require care from multiple specialists. On the ICW a daily treatment meeting is held between multiple specialists (internist, geriatrician, pulmonologist, cardiologist, and hospitalist) to collectively discuss treatment options and provide patient centred care. Therefore, the ICW meeting was chosen as the IP meeting type. The hospitalist is the discussion leader and contact person for the patients. Sometimes trainees such as residents, pharmacists or nurse specialists attend these meetings for educational purposes.

More detailed information regarding the procedures of the ICW has been described in previous publications [20,24].

The endocarditis meeting was considered to be an MP meeting because professionals work alongside each other and focus on their own speciality/organ. The endocarditis meeting discusses patients suspected of having endocarditis or being treated for endocarditis and is held on a weekly basis to formulate a treatment plan. The meeting usually consists of at least one infectious disease medical specialist, one microbiologist, several cardiologists, and several residents and medical students.

### Data collection procedure

Data was collected by video and audio recording, allowing researchers to assess and observe individual behaviours and interactions in a natural environment without direct physical intrusion [25]. A researcher (RR) set up the recording equipment in the room before to the meetings to limit the impact of the observations. Immediately after the meeting was recorded, the recording was anonymized by placing a ‘beep’ over any data that could identify participants or patients. After anonymisation, the recordings were transcribed verbatim by an independent typist who was not part of the study group. Raw transcripts were read and, in cases of ambiguity, the original video and audio observations were used to correct and complete the transcripts by MH, RR and SdG.

### Data analysis

Firstly, general characteristics of the meetings and participants were summarised, and the setting was visualised.

**Fig 2:**
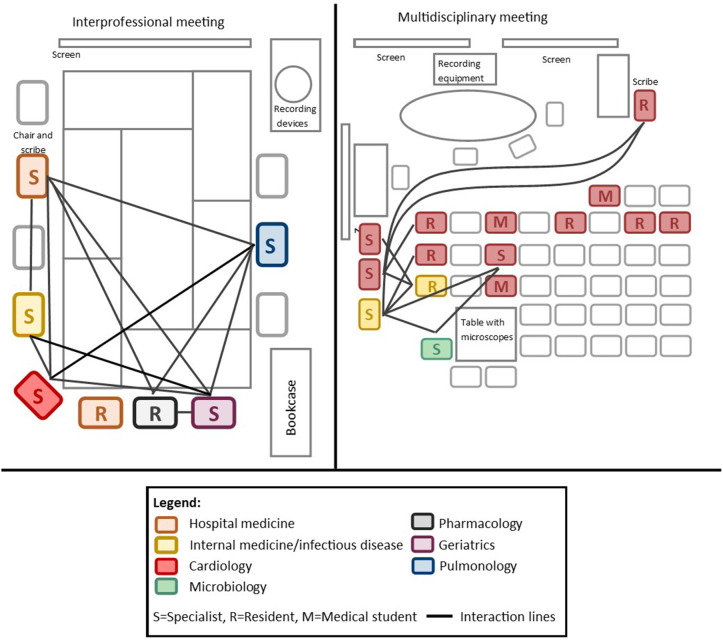
Seating arrangement and interaction diagrams in Interprofessional and multidisciplinary meetings.

Then, to collect the qualitative research results, the audio and video recordings were analysed in several steps. The method to do so was an inductive conventional content analysis combined with thematic analysis [26]. Conventional content analyses is an inductive method in which themes are created from textual data instead of from pre-existing theories [27]. With conventional content analysis, patterns can be identified. Subsequently, a thematic analysis was used to identify themes within the data [28]. To take all these steps, all documents were uploaded into Atlas TI, a software programme for computer assisted analyses in qualitive research. Fig 2 shows the different steps taken.

In the first step, five researchers (SdG, RR, MH, CK and NL) developed an observational focus and framework based on Spradley’s nine observation dimensions [29], see S1 Table. In the second step, the researchers validated this observation focus by independently observing the first two video recordings and then discussing the results together. In the third step, three researchers (SdG, RR, MH) independently observed the other video recordings and took fieldnotes using the observation focus.

In the fourth step, three researchers (CK, SdG and MH) analysed the first transcript together, resulting in an initial coding template. In the fifth step, all transcripts and fieldnotes were coded using an open coding approach followed by axial coding. All transcripts and fieldnotes were coded by at least two researchers (SdG, RR, MH) to reduce observer bias. In case of discrepancies between codes, the researchers discussed and resolved these differences together. The three researchers agreed that after a total of 10 meetings (5 of each type of meeting) no new themes or codes emerged in analysis and thus data saturation was reached. In the sixth step, themes were developed and discussed with the research team. In this session, facilitators and barriers to effective collaboration were identified and recommendations for improving collaboration were developed.

Finally, to provide an additional perspective, three researchers (SgD, RR, MH) assessed the meeting performance using the validated MDT-OARS (Multidisciplinary Team – Observational Assessment Rating Scale), as found in the literature [30,31]. The MDT-OARS scores 15 areas of meeting performance in four categories. The scores range from 1 to 4, very poor to very good, and are based on whether predefined criteria were met or not. Mean scores were calculated for each type of meeting. S2 Table shows the MDT-OARS pre-defined criteria in detail. The MDT-OARS score was the most appropriate, as it best represents the facilitators and barriers to effective collaboration identified in previous literature.

### Reflexivity

The research team consisted of members from a range of backgrounds to include different perspectives. All team members have experience with interprofessional collaboration and/or research.

CK is a geriatrician, clinical pharmacologist, dean of interprofessional education and one of the co-founders of the ICW. She attends some of the ICW meetings. NL is a psychologist with experience in interprofessional collaboration and education. SdG and RR are PhD students and doctors with work experience in a geriatric unit, SdG also represented the patient perspective as he has a chronic illness. MH is a medical student. SdG and RR’s supervising teams were also involved, consisting of two geriatricians (HK, ME), a general practitioner who is also a professor of medical education (MvdP), and a clinical epidemiologist (BvdZ).

### Ethics

Healthcare professionals were observed in their daily working environment without any intervention other than recording. The physical and psychological integrity of the participants and the patients discussed was maintained throughout this research. The local Medical Ethical Review Board (METC) declared this study to be outside the scope of the Medical Research Involving Human Subjects Act (WMO) (METC number NW2023−01). Written informed consent was obtained from all participants before recording. They were allowed to withdraw from the study at any time. This study was conducted in accordance with the tenets of the Declaration of Helsinki.

## Results

### General results and observed participants

Five multidisciplinary (MD) meetings and five interprofessional (IP) meetings were recorded between June and August 2023. The characteristics of both meeting types are described in Table 1. Notably, more professionals attended the MD meetings than the IP meetings but in the IP meetings a greater diversity of specialties was represented. Five participants attended both meeting types (1 infectious disease medical specialist, 3 cardiologists, 1 cardiology resident). The average discussion time per patient was similar in both meeting types.

**Table 1:** Details of the treatment meetings and participants.

	*INTERPROFESSIONAL (IP)* Intensive Collaboration Ward	*MULTIDISCIPLINARY (MD)* Endocarditis meeting
Number of observed meetings	5	5
Mean number of patients discussed per meeting (range)	6 (2-8)	4 (3-6)
Mean duration of meeting in minutes (range)	28:54 (08:08-41:42)	19:37 (14:40-27:36)
Mean discussion time per patient in minutes	4:38	4:39
Number of professionals per meeting*		
Number of professionals per meeting (range)	5-7	12-15
Internal medicine/ infectious disease	1	0-1
Geriatrics	1	0
Hospitalist	1	0
Cardiologist	1	1-5
Pulmonologist	1	0
Microbiologist	0	1
Residents**	0-1	4-8
Medical student	0	1-3
**Participant characteristics**		
Mean age	40 (28-55)	35 (23-57)
Sex per meeting (m/v)	1-4/2-6	3-5/8-11

* 1 Internal Medicine Specialist, 3 Cardiologists, 1 Cardiology Resident attended both meetings.

** At multiple IP meetings, a nurse specialist and a pharmacist attended for educational purposes.

### The themes

Four main themes emerged from the analysis: 1. Setting and surrounding, 2. Patient perspective, 3. Interaction between healthcare professionals, 4. Contribution of individual healthcare professionals. From these themes, and their subthemes, nine recommendations were formulated, which will be referred to as the nine keys to successful collaboration. The themes, sub-themes and coding template are available in S1 Fig.

1. Setting and surrounding
  1.1 Seating arrangements

**Fig 3:**
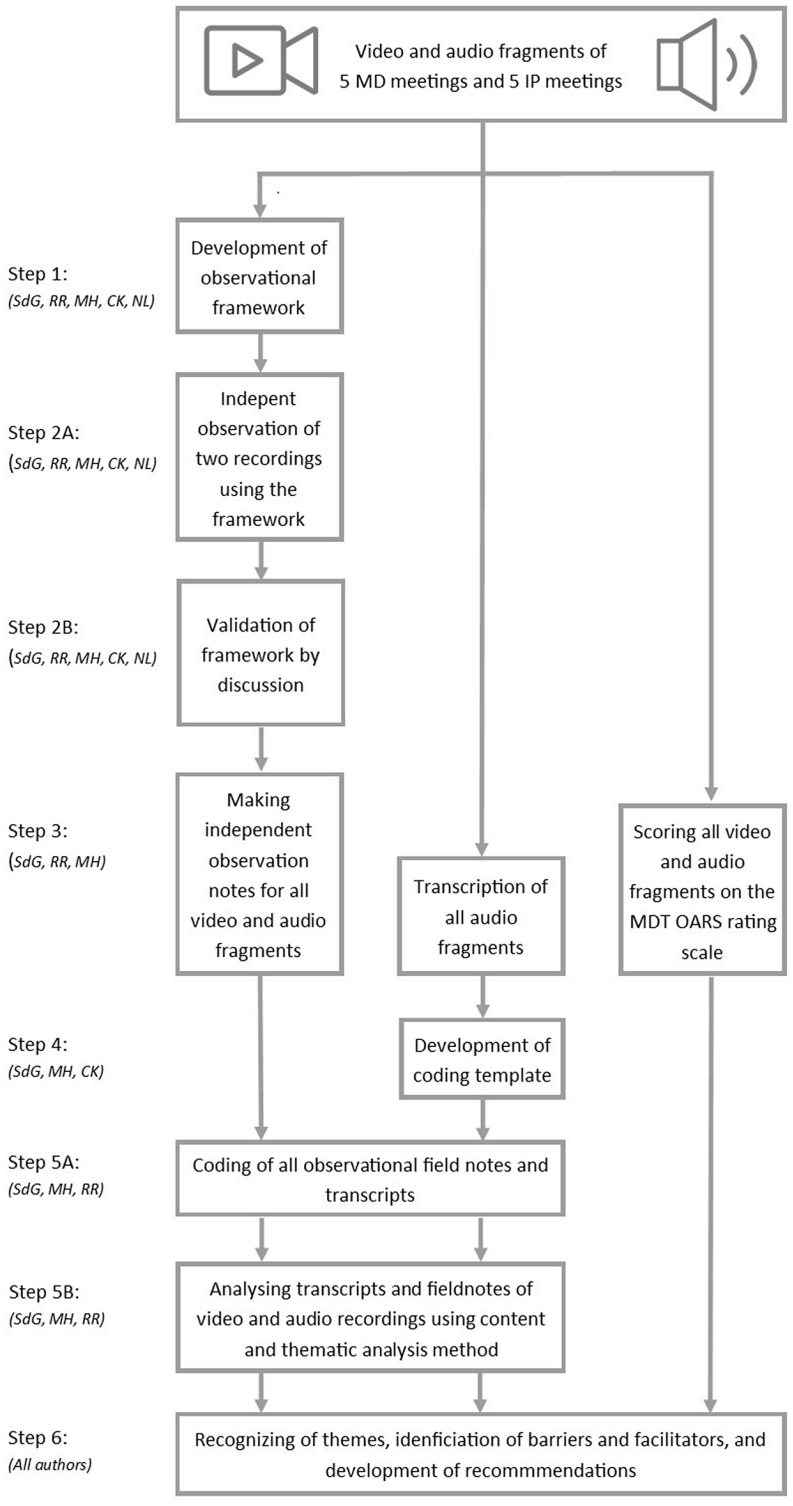
Overview of the different steps taken in the data analysis process. MD = Multidisciplinary. IP = Interprofessional.

Fig 3 illustrates the different seating arrangements of the two meeting types: the MD meeting used a theatre arrangement with participants facing each other’s backs, while the IP meeting used a round table arrangement. The large theatre arrangement allowed participants to leave chairs empty between each other, resulting in a greater physical distance between participants. Furthermore, participants seemed to cluster according to specialty and background. Infectious disease and microbiology physicians sat in the back, cardiologists sat on the side of the room, and residents and medical students filled the front rows. There was no particular seating arrangement during the IP meetings.

These differences seemed to affect the individual engagement of participants. In the MD meetings, the percentage of participants who engaged in the central discussion ranged from 50–79%, whereas in the IP meetings this ranged from 86–100%. We observed that in the MD meetings, most of the discussion took place between the cardiologists and the infectious disease medical specialist, who sat at the back and left of the room. Participants in the front rows were often not involved in the central discussion. They mostly looked at the screen in front of them while the discussion took place behind them. They also yawned, looked out of the window, or whispered to each other during the central discussion. During the IP meetings, we observed that participants were more likely to face each other rather than look at the central screen, and that participants who contributed less verbal information regularly took notes. These dynamics are illustrated in Fig 3.

1.2 External distractions

In both types of meeting, distractions seemed to hinder the effectiveness of the discussion by preventing one or more participants from engaging in the central discussion. The level of external distraction was similar in both meeting types. These distractions mostly consisted of mobile phone alerts, participants rejecting calls or leaving the meeting. During one of the MD meetings, participants were also distracted by workers outside the window.

2. Patient perspective
  2.1 Patient centeredness

In the IP meetings, the patient was the central focus of the discussion, with emphasis on their perspective, social context, and functional abilities. These aspects were typically introduced by the hospitalist, who had direct knowledge of the patient’s perspective through regular face-to-face interactions. The following quote highlights the hospitalist’s familiarity with the patient’s functional abilities and social environment.

*IP Hospitalist: “…. Also this man is in need of care, as in part of the instrumental daily activities have been taken over by his daughters [and] he receives homecare twice a day for helping with putting on and taking off his compression socks. Well, socially; [he has] fifteen rabbits. I always copy it [information on social context from the emergency notes] and extract relevant things, but this [having 15 rabbits] I found relevant (laughter).”*

In contrast, the MD meetings focused on medical information and often lacked information about the patient’s context or perspective. The following quote shows how patients are introduced in the MD meeting:

*MD infectious disease resident: “... has a history of mitral valve replacement, a bioprosthetic and a tricuspid valve repair and in 2022 a VVI pacemaker implantation. He is admitted with a culture negative endocarditis. …”*

During the IP meetings, the patients’ prognosis was explicitly discussed seven times. During the MD meetings, prognosis was discussed once. Below is a quote from a discussion of prognosis during the IP meeting.

*IP Cardiologist: Yes, but for the short term the prognosis is determined by the wobbly under/over fluid status. He probably has a severe aortic valve stenosis for which we have no treatment options. Therefore, I am a bit pessimistic [about his prognosis].*

2.2 Proportionality of care

In the IP meetings, participants anticipated possible test results and discussed whether new findings might affect the treatment plan. In the MD meeting, diagnostic tests were sometimes performed (e.g., an echocardiogram), but regardless of the results, the treatment plan was often primarily influenced by the patient’s clinical condition. Box 1 illustrates two discussions on the value of diagnostics from the IP and MD meetings.

**Box 1: Discussion on value of diagnostics**

**IP meeting:**

*Geriatrician:I think an ultrasound is definitely indicated with such levels of gamma-glutamyltransferase and alkaline phosphatase.*

*Internist:With the consequence that if a cholecystitis is confirmed, he needs the right antibiotics, but if we want to know if there is an [bileduct] obstruction that he needs desobstruction.*

*Geriatrician:I think that if the [potential] obstruction looks like a stone or like a tumor there are different courses of action. He might be fit enough for a stone desobstruction but if there is a malignancy with potential more [future] crisis, you could question…. [how this impacts current treatment options].”*

***Situational description:***

*Before ordering an additional test (ultrasound) participants evaluate whether the result of the test would*

*affect treatment choices.*

***MD meeting:***

*Cardiologist while evaluating frames of a transthoracic echocardiogram:*

*‘Well, this is no super quality. But at least we don’t see any leakages on this level and the valves look, as far as it is assessable, normal. Go to the apical frames. If you…. Exactly. This is very poor [image quality] Yes. This is really… Yes, very poor image quality. So this echocardiogram, you can barely call it diagnostics.’*

*Cardiac resident: ‘That is what we expected beforehand.’*

*Cardiologist:‘Well you can say, no irregularities, however the image quality is so poor… And what did we plan to do’ [if the quality was so poor]?*

*Cardiac resident:‘That we would not perform a transoesophageal echocardiogram, because actually…’[the probability of endocarditis was low]*

*Cardiologist:‘so this is it?’*

*Cardiac resident: ‘yes and the patient is clinically improving.’*

**Situational description:**

After stating the results of the performed diagnostic test (transthoracic echocardiography) were not reliable

the decision was made to not perform another more reliable test (transesophageal echocardiography)

because the pretest probability for endocarditis was low anyway and the patient improved clinically. So, in

hindsight the performed transthoracic echocardiogram served no purpose.

2.3 Discharge planning

In the IP meetings, discharge planning was discussed from the patient’s perspective. In the MD meetings, discharge planning was about dividing tasks and practical planning. Below is a quote from the MD and IP meetings about planning follow-up appointments. In the MD meeting, the logistical challenges from the doctor’s point of view are highlighted.

*MD infectious disease specialist: ‘Yes. I think you have to organize this well. Perhaps… Is that something you would want to take a leading role in? Or are you unavailable for it in the coming time?’*

*MD infectious disease resident: Well, this is my final week as a consultant, after that I have a week of night shifts and then I will be back for only one week. For me it is not convenient, I won’t be around much.*

In contrast, in the IP meeting, doctors try to limit the number of future appointments with different doctors to accommodate the patient’s perspective, as the following quote shows.

*IP geriatrician to pulmonologist: ‘Do you want to see her again? Since you also listen to the lungs, if you hear any fluid there, maybe you can adjust the diuretics [cardiac medication] before she ends up in three places. We will wrap up the cognition [analysis] before discharge, so she doesn’t need an appointment with us.’*

So instead of having an appointment with a pulmonologist, cardiologist and geriatrician, the patient now only has an appointment with the pulmonologist.

3. Interaction between healthcare professionals.
  3.1 Team composition and individual roles

We observed that an unbalanced team composition may affect individual contributions to the central discussion. As described in Table 1, the IP team was smaller (average number of participants 5–7), and each specialty was represented by one or two doctors. All participants had a role in the discussion, namely, to contribute input from their respective specialty. No two participants had the same role. This seemed to encourage individual contributions from the participants, as specific information could only be contributed by one participant.

In contrast, the MD team was larger (average number of participants 12–15) and consisted mainly of cardiologists. Cardiologists outnumbered infectious disease and microbiology physicians by a ratio of 3:1–2:1. The cardiologists all had similar roles, which resulted in some cardiologists contributing more to the discussion and others leaning back. In addition, the proportion of residents and medical students was higher in the MD meeting than in the IP meeting. Only a small proportion of the residents and medical students took part in the discussion, such as presenting the patients or interpreting the echocardiogram. A large proportion of participants were less involved and did not contribute to the discussion or ask educational questions.

3.2 Predesignated tasks: chair, patient introductions, and scribe

The effectiveness of the treatment discussion seemed to improve when there was a pre-determined chair. It also appeared to be beneficial when the person introducing the patient also formulated a central discussion point. In the IP meetings, the chair was always the same person, the hospitalist. They introduced the patient in a standardised format and ended with a central discussion point. In the MD meetings, there was no pre-determined chair, and the discussion was led by the person introducing the patient (a cardiology or internal medicine resident) or the by the specialist most familiar with the case. Sometimes no doctor was prepared to present the patient, resulting in less coherent patient presentations without a clear point of discussion. The following quote confirms this observation.

*MD cardiologist to resident internal medicine: ‘It’s nice that you introduce all of our patients…’ Resident internal medicine: ‘Yes, … but if we don’t know who the [treating] physician is, I’ll share what I know and then we will see what else you all know.’*

Using the MDT-OARS scores, we found that the conclusion was more clearly stated in the IP meetings compared to the MD meetings. In the IP meetings, the conclusion was summarized at the end of the discussion by the hospitalist or another participant, while the hospitalist documented it in the patient’s record. In the MD meetings, however, the conclusion was only summarized for the scribe if specifically requested. As the scribe was a cardiology resident sitting at the other end of the room, he often had to interrupt the discussion to ask what should be documented in the medical record. The frequent interruptions seemed to lead to inefficient discussions.

3.3 Atmosphere

Our observations showed that a positive atmosphere seemed to foster non-hierarchical communication and increase individual engagement.

The IP meetings were characterized by a relaxed atmosphere, with open interaction, humour, laughter, and compliments. Almost all participants, regardless of their role or background, actively contributed to the discussion. Occasional redirection was necessary to keep the focus on the patients and to manage the time effectively. The average length of discussion per patient was similar in both types of meeting (see Table 1).

The atmosphere at the MD meetings was not as relaxed and the interaction exhibited a more hierarchical style. Trainees and residents from the cardiology department often did not contribute to the meeting. Sometimes the atmosphere felt tense and participants seemed to disagree more often and there were fewer jokes. The following quote shows a specialist correcting a resident during the main discussion, without clear reasoning or explanation. At this moment, the researchers observed a tense atmosphere, which is documented in the observation notes.

*MD Specialist internal medicine to resident cardiology: ‘…So, we really did execute the work-up well, that’s important to mention. It doesn’t actually seem like it now.’*

*Resident cardiology: ‘No, no, no, there was nothing to see on all the tests.’*

*Specialist internal medicine: ‘No, but that’s important to mention.’*

After treatment discussions, tasks were divided between participants. In the IP meetings, the division of tasks was more often formulated as a question and the communication felt non-hierarchical. The following quote illustrates how a cardiac resident is encouraged to carry out the outpatient follow-up of a frail elderly heart failure patient during one of the IP meetings.

*IP hospitalist to a cardiac resident: ‘Will he come back to you for follow up?’*

*Cardiac resident: ‘Well, I will be doing outpatient clinic, so I could do the follow up.’*

*IP Cardiologist: ‘Yeah just do it!’*

*IP Geriatrician: ‘Yes nice!... If that isn’t intensive collaboration ward-like!’*

*Cardiac resident: ‘Yea right! This patient was made for me.’*

In contrast, in the MD meetings, tasks were formulated as assignments given by specialists to residents. Communication seemed to be more hierarchical. In addition, residents sometimes seemed surprised or uncomfortable after receiving a task from their supervisor. However, they did not explicitly express their discomfort. In the following quote from an MD meeting, a resident is asked to call a patient to the emergency department, although the resident does not feel comfortable doing this, the cardiologist insists that he does it anyway.

*MD cardiologist to resident cardiology: ‘I think someone needs to invite him to the Emergency Department…’*

*Resident cardiology: ‘The way this is going seems a bit odd to me, because the man hasn’t raised any alarm himself. Should I call him, hearing indirectly that he’s feeling a bit short of breath?’ … ‘I don’t know the man at all…’*

*Cardiologist: ‘That doesn’t matter.’*

At both meetings, participants expressed feelings of insecurity, doubt, and concern for the patient. They also showed their vulnerability by admitting a mistake or lack of knowledge. The next two quotes from both types of meeting illustrate expressions of vulnerability.

*MD microbiologist: ‘The point is, and that’s apparently my own negligence, but he isn’t getting enough amoxicillin.’*

*IP hospitalist: ‘They wanted him at home. I feel… Did I do something wrong, did I let him go home too early?’*

Participants in the IP meetings showed trust in each other, even when it came to their own specialty. For example, the geriatrician, who is a specialist in cognitive diagnostics, tells the hospitalist that it is up to the hospitalist’s clinical judgement whether a cognitive screening test (a Montreal Cognitive Assessment (MOCA)) is indicated.

*IP geriatrician to hospitalist: ‘Very good. [You can] evaluate if you still need a MOCA.’*

3.4 Interprofessional learning

There are three levels of IP learning: learning with, from and about each other [32]. Our data showed that all three levels of IP learning took place during the IP meetings. We observed that all participants in the IP meetings, regardless of specialty or function, asked questions about topics outside their specialty and spontaneously shared knowledge. The following quote illustrates a moment during the IP meeting when participants learn from each other.

*IP geriatrician to pulmonologist: ‘What do you see at first glance?’ After this question, the pulmonologist explained how to read a spirometry.*

Participants in the IP meeting also learned with each other. The following quote shows one participant reminding the others that they must remain critical and also evaluate the indication and benefit of a lipid lowering drug (statin) in an older patient population.

*IP specialist internal medicine to hospitalist: ‘But this is the ICW (IP meeting), we stop, we don’t prescribe statins?’*

The next quote from the IP meeting illustrates a moment of reflexivity and empathy with the previous doctor who had apparently missed a diagnosis, but also a moment when participants could learn from and about each other.

*IP geriatrician: ‘If we go back all the way to the beginning of the story, looking backward it’s always easy to judge, but there’s a man with a lot of back pain, so much pain that he had to go to the rehabilitation centre. Back pain is not a diagnosis, right?’*

In contrast, learning with, from and about each other seemed to occur less frequently in the MD meetings. There were fewer educational questions and less spontaneous knowledge sharing in these meetings. Although the MD meetings should also function as an educational moment for residents and medical students. In addition, in the MD meetings the residents sometimes seemed hesitant to ask a question and even apologized in advance. This was not observed in the IP meeting. This first quote illustrates a resident’s hesitation to ask an educational question.

*MD resident internal medicine: ‘Probably a stupid question, but just so I know…’*

The next quote illustrates how a resident is trying to introduce a learning opportunity for himself and others, but the consultant is postponing this teaching moment because the specialist found that it was not the right time.

*MD resident internal medicine on differential diagnosis of a culture negative endocarditis:*

*‘We have completed all the diagnostics. However, I did research some tests of which I thought [maybe we should consider them], but…’*

*MD infectious disease specialist: ‘You can ask me later.’*

3.5 Listening

Our observations showed different levels of listening: bad-, pretend-, selective-, attentive- and empathic listening. Attentive listening occurred in both types of meeting but was more present in the IP meetings. The IP meetings showed several examples of empathic listening, where participants seemed to really try to understand the other participant’s point of view. On the contrary, selective listening and bad listening were more present in the MD meetings. The following quote illustrates how two specialists interrupt each other and discuss different topics without listening to each other.

*MD Specialist internal medicine: ‘But you could discuss adding doxycycline empirically while waiting on further diagnostic tests, I think...’*

*MD Cardiologist: ‘I think we need to look at the echo.’*

4. Contribution of individual healthcare professionals
  4.1 Active participation and stepping outside one’s own specialty

We found differences in the participation of individual healthcare professionals between IP and MD meetings. In the IP meetings, all professionals participated actively, contributing their expertise to the discussion, asking questions outside of their own specialty, and trying to argue and explain their thoughts. Even if they were the specialist on the topic, they asked for input from others.

On the other hand, in the MD meetings, a significant part of the team, especially medical students and residents, were not actively involved. Also, there was often someone who left early, whereas no one left at the IP meetings. In addition, specialists tended to stay within their own specialty and were less likely to ask questions outside their own specialty. The following quote illustrates a pulmonologist sharing a thought on the differential diagnosis of renal decline.

*IP Pulmonologist: ‘Can you get a septic embolism with endocarditis as the cause of the [decline of] kidney function?’*

The following quote illustrates a participant asking a question about his own area of expertise as a form of teaching.

*IP Cardiologist: ‘What are we going to prescribe for her rate control?’*

4.2 Behavioural differences of participants that participated in both meeting types.

In total, five doctors participated in both the MD and IP meetings. We observed a distinct variation in the behaviour of doctors who participated in both meetings. At the MD meetings, all five participants asked fewer questions about the patient’s perspective and did not engage in interprofessional learning. Conversely, at the IP meetings, the same participants spontaneously provided information or asked questions for educational purposes. They actively participated in discussions and stepped outside their specialty. In particular, one cardiology resident did not ask any questions, only looked at the screen in front of her and did not participate verbally or non-verbally in the discussion throughout the MD meeting. During the IP meeting, she actively participated by asking educational questions and contributed to a positive atmosphere by laughing and making jokes. Here are some quotes that support these observations from the IP meeting.

*Quote 1:IP cardiac resident about a patient with cholangitis: ‘Isn’t this someone that if he does not improve, he could benefit from ERCP (endoscopic retrospective cholangiopancreatography)?’*

*Quote 2:IP geriatrician about a heart failure patient: ‘And, to patients with HFpEF (heart failure with preserved ejection fraction) you give diuretics and ace inhibitors, right?*

*IP cardiac resident: ‘[You give] an SGLT2-inhibitor. (Sodium-Glucose-transport-protein 2 inhibitor = heart failure medication)’*

### Team performance by MDT-OARS assessment tool

Beside above themes, the team performance of the meetings were scored by a validated scoring system to measure team performance. The mean MDT-OARS for each type of meeting are shown in Table 2. IP meetings had higher mean scores than MD meetings for the categories ‘teamworking and culture’ (20 (19–20) vs 12 (10–14)) and ‘clinical decision making’ (6 vs 3 (2–5)). Other categories had similar scores.

**Table 2:** Meeting performance as measured by MDT-OARS mean scores and range per domain.

The category ‘teamworking and culture‘includes the inclusion of team members, team sociability, mutual respect, and tension and conflict. The category ‘clinical decision making’ includes patient-centred care and treatment plans.

## Discussion

**Fig 4:**
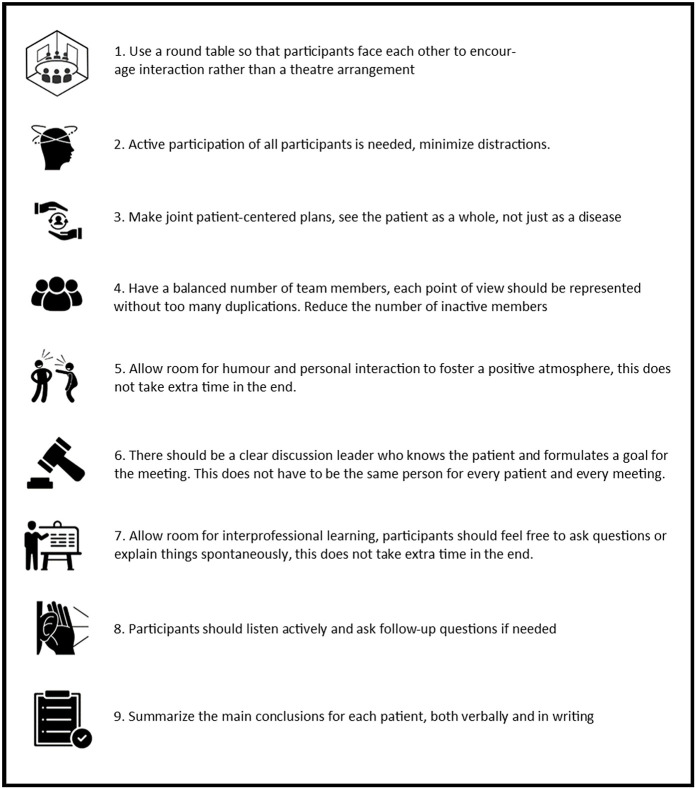
Nine keys to successful collaboration.

This study aimed to investigate the differences between interprofessional (IP) and multidisciplinary (MD) meetings by observing two meeting types with comparable case complexity and similar participating specialties. Four key themes emerged, each demonstrating distinct differences: 1. Setting and surrounding, 2. Patient perspective, 3. Interaction between healthcare professionals, 4. Contribution of individual healthcare professionals. Our findings showed that IP meetings were more patient-centred, fostered a more relaxed and positive atmosphere, and provided a better environment for interprofessional learning. Based on our data, we formulated nine keys to effective collaboration, see Fig 4.

The behaviour of individuals and the interactions between them differed between the two meeting types, as illustrated in Fig 3. In the MD meetings, participants generally contributed less to the treatment discussion, asked fewer educational questions, and the atmosphere seemed more tense. In the IP meetings, the opposite appeared to be true. These differences in behaviour might be explained by differences in intrinsic values and preferences among clinicians participating in the different meetings. Some clinicians may be more inclined to collaborate or to make patient-centred plans. However, as we did not perform in depth interviews with participants regarding their motivations or preferences, we do not know whether there are any differences between clinicians of both meeting types. Notably, some participants attended both meeting types yet demonstrated markedly different behaviors across the two settings. This suggests that external contextual factors may either support or inhibit physicians’ intrinsic motivation to collaborate. Indeed, even individuals who remained disengaged during the MD meetings showed a high level of interaction in the IP meetings. In fact, among the participants who attended both meeting types, even those who did not engage during the MD meetings exhibited a high level of interaction during the IP meetings.

One explanatory factor for this phenomenon is the presence of power dynamics. In the MD meetings, the cardiology physicians outnumbered the infectious disease and microbiology specialists, and a pronounced hierarchical structure was evident, with medical specialists speaking more frequently than residents and occasionally even interrupting them. These two factors, the overrepresentation of a single specialty and a strong, potentially dysfunctional, hierarchical structure, are known to limit the participation of healthcare professionals [33]. When designing treatment meetings, it is important to make such implicit dynamics explicit (e.g., by discussing them) to create a culture that encourages active participation. While hierarchy can serve a functional role in interprofessional collaboration and learning, it can also become counterproductive if left unaddressed. This reduced participation of healthcare professionals is also related to some other factors described in this study, such as allowing room for humour and fostering a positive atmosphere and interprofessional learning. Reducing the overrepresentation of one specialty and dysfunctional hierarchies may also improve these elements and contribute to more inclusive and effective meeting dynamics.

Another factor that can influence the interactions and behaviour of individuals is the setting. For example, the IP meetings used a roundtable setting where participants could see each other, which encouraged interaction. The MD meetings used a theatre setting where participants could not see each other, which could hinder interactions and change individuals’ behaviour (Fig 3). This should be considered when designing a collaboration.

Unconstructive power dynamics can also negatively influence learning [34]. This is supported by the results of this study, which show minimal IP learning in the MD meeting and extensive IP learning in the IP meeting. Workplace learning is essential for residents and medical students (learners), and designing treatment meetings to promote IP workplace learning enables learners to develop. Then learners and professionals can learn with, from and about each other every day in their daily work. Currently, most medical education curricula do not optimally prepare students for interprofessional collaboration in clinical practice, despite previous research describing the benefits of such collaboration [35–36].

The positive atmosphere of the IP meetings fosters individual participation and learning, and it stimulates jokes and detours. Our research shows that humour and jokes do not lead to longer, inefficient meetings, as the discussion time per patient is similar between the two types of meeting. Previous research already established that laughing together can have a positive impact on the wellbeing of participants and the team climate, and it promotes the delivery of team-based care [37]. This may explain why IP participants more frequently listen to each other attentively, which enhances the effectiveness of communication during the meeting. Participants in MD meetings listen more selectively or not good at all, and they disagree with each other more often, which can be time consuming [38].

The preliminary results of this study were presented to more than 60 healthcare professionals, including medical specialists, residents, and educationalists, at a major national scientific congress in the Netherlands. They agreed with the findings of our study and recognised them from their own clinical practice, with some professionals already implementing some of the key messages. For example, one professional noted that they were currently implementing round-table settings. One participant questioned whether certain behaviours were inherent to certain individuals. When conducting this research we had the same presumption, however, our findings demonstrated that five participants exhibited markedly different behaviours in the two different meetings. The reactions of the professionals at the congress further confirmed our findings. In addition, the findings of our study were presented to the cardiologists of the MD meeting investigated in this study. They had already recognised the need for improvement, but were unsure about the areas requiring change and how to implement it. An educationalist is currently supporting them in implement improvements.

### Strengths and limitations

To the best of our knowledge, this is the first study to compare IP meetings with MD meetings and to identify the facilitators and barriers to collaboration in both settings. Although many facilitators and barriers to collaboration have been documented in the literature, these have focused on a single type of meeting. By comparing two meetings with similar participants and patient cases, a direct comparison can be made, and key differences can be highlighted. This study has several strengths. First, the triangulation within the research team; this study brought together an interprofessional research group with different professional and personal backgrounds and training, which provided diverse perspectives into the observations and analysis of the data. Second, this study provides practical recommendations for improving patient care meetings that is widely applicable and transferable to different types of meetings.

This study also has limitations that should be considered. First, the IP and MD meetings observed in this study may not be representative of all IP and MD meetings that occur. Therefore, our findings regarding the occurrence of facilitators and barriers to collaboration may not be generalizable to all IP and MD meetings. However, the identified keys to effective collaboration are not specific to one type of meeting but may positively influence various types of treatment meetings involving older multimorbid patients. Second, observer bias could affect the objectivity of the researchers. To minimize this bias, three researchers independently observed and analysed the data. In addition, the use of a structured observation framework improved inter-rater reliability. Third, participants’ awareness of being observed could influence their behaviour, so we chose non-participant observations. In addition, to minimize participants’ awareness of being filmed, the cameras used to film the sessions were pre-existing cameras integrated into the main screen. Finally, the study relies solely on audio and video observations, which limits insight into participants’ thoughts or behaviour. Future research should consider using interviews or focus groups to gain insight into participants’ perspectives.

## Conclusion

This study showed that several factors may influence collaboration and participants’ behaviour related to active participation, learning, and patient-centred care in both interprofessional (IP) and multidisciplinary (MD) meetings. Factors such as a round-table setting, a designated chair, discussions focused on a shared patient-centred goal, active engagement from participants, and a relaxed atmosphere appeared to support team collaboration and interprofessional learning. Moreover, the differences in the behaviour of the same participants across the two meeting types further suggest that these contextual factors can shape participant behaviour. Based on these findings, we developed nine key strategies for optimising collaboration that might improve collaborative practice.

## Supporting information

S1 Table Observation focus, based on Spradley’s nine observational dimensions. (DOCX)

S2 Table MDT-OARS. (DOCX)

S1 Fig MDT-OARS. (DOCX)
