# Supplementary material for: Nine keys for successful interprofessional collaboration Based on observing facilitators and barriers during different types of treatment meetings: A qualitative study
Source: PLoS One. 2026 Jul 1;21(7):e0350554. doi: 10.1371/journal.pone.0350554 (PMC13322501; doi:10.1371/journal.pone.0350554)
Supplement: S1 Table — (DOCX) [file pone.0350554.s001.docx]

**Supplement 1 Table. Observation focus, based on Spradley’s nine observational dimensions**

| **Dimensions** | **Descriptor** | **Example of questions** | **Video observation** | **Audio transcriptions** |
| --- | --- | --- | --- | --- |
| Space | Physical layout of the place | How would you describe the environment? | X |  |
| Actor | Participant characteristics | Who collaborates with whom?  Which actors are positive and negative role models and why? | X |  |
| Activity | A set of related activities that occur | What are the critical factors influencing the actors?  What activities take place during the meeting? | X | X |
| Object | The physical things that are present | What do the actors see?  Do actors and observer see the same physical things? | X |  |
| Act | Single actions people undertake | What are the actions of the actors?  What are actors participating in?  What would actors like to do? | X | X |
| Event | Activities that people carry out | How do actors address interprofessional aspects during the meeting? | X |  |
| Time | The sequencing of events that occur | What happens first, what happens after, etc.  When do moments of collaboration arise in the meeting?  Do actors explicitly label interactions as collaborative? | X | X |
| Goal | Things that people are trying to accomplish | Have common goals been established among the actors?  Are the goals aligned with one another? | X | X |
| Feeling | Emotions felt and expressed | Have actors expressed emotions?  Are there situations where actors are individually addressed regarding their professional roles? | X | X |
| Observer | Emotions of the observer | What emotions does the observer experience during the meeting? | X | X |

An X indicates if this dimension was reported on using the video observation and/or audio transcription.
