# Supplementary material for: Nine keys for successful interprofessional collaboration Based on observing facilitators and barriers during different types of treatment meetings: A qualitative study
Source: PLoS One. 2026 Jul 1;21(7):e0350554. doi: 10.1371/journal.pone.0350554 (PMC13322501; doi:10.1371/journal.pone.0350554)
Supplement: S2 Table — (DOCX) [file pone.0350554.s002.docx]

**Supplement 2 Table. MDT-OARS**

| **Characteristic of effective MDT working** | **Quality Criteria** | **Quality of team working (score in brackets)** | | | | |
| --- | --- | --- | --- | --- | --- | --- |
|  |  | **Very Poor** | **Poor** | **Good** | **Very Good** | |
| **The Team** |  |  | | | | |
| Attendance | Presence of relevant core team members at the meeting | at least one core team member (and their deputy) is not present for the whole meeting | at least one core team member (and deputy) is absent for most of the meeting (≥3 cases) | at least one core team member (and deputy) is absent for part of the meeting (≤ 2 cases) | all core team members (or deputy) present for whole meeting | |
|  |  | (1) | (2) | (3) | (4) | |
| Leadership: chairing of meeting | · Keeps meeting to agenda (i.e. moves onto next case) | Satisfies none of criteria | Only satisfies 1–2 of criteria | Satisfies 3 of criteria | Evidence of all of the criteria | |
|  | · Encourages overall participation |  |  |  |  | |
|  | · Encourages focussed discussion |  |  |  |  | |
|  | · Articulates recommendation | (1) | (2) | (3) | (4) | |
| Teamworking & culture | | | | | | |
| a) Inclusion of relevant team members | · All relevant core members are actively and appropriately involved | Satisfies 1/none of criteria | Satisfies 2–4 of criteria | Satisfies “*all relevant core members are actively and appropriately involved*” and at least 3 other criteria | Satisfies all of the criteria | |
|  | · Meeting not dominated by 1-2 people |  |  |  |  | |
|  | · Input/questions volunteered and encouraged |  |  |  |  | |
|  | · Contributions facilitate decision-making and/or inform discussion |  |  |  |  | |
|  | · Consensus of decision-making | (1) | (2) | (3) | (4) | |
| b) Team Sociability | · Evidence of humour | Satisfies none of the criteria | Satisfies 1 of criteria | Satisfies 2–3 of criteria | Satisfies all of criteria | |
|  | · Team appear relaxed with each other |  |  |  |  | |
|  | · Warm and supportive team environment |  |  |  |  | |
|  | · Friendly and cooperative communicative style | (1) | (2) | (3) | (4) | |
| c) Mutual respect | · Focussed attention | Only satisfies 1 or none of | Satisfies 2–3 of criteria | Evidence of respect, | Strong evidence of | |
|  | · Respect for speaker | criteria |  | evidence of at least 4 | respect in all/almost all | |
|  | · No concurrent discussions |  |  | criteria | cases | |
|  | · Asking and valuing relevant contributions |  |  |  |  | |
|  | · General sense of politeness/courtesy (inc mobile phone etiquette) | (1) | (2) | (3) | (4) | |
| d) tension and conflict |  | *Not rated on the same scale – see bottom of table* | | | | |
| Personal development & training | Observable communication of research evidence and/or instances of learning | No observable communication of research evidence or instances of learning | Minimal communication of research evidence or instances of learning | | Structured presentation of research evidence and/or learning through formal discussion (e.g. of audit findings | |
|  |  | (1) | (2) | | (3) | |
| **Infrastructure for meetings** |  |  | | | | |
| Meeting venue | · Room size appropriate for number of team members | Satisfies only 1 or none of criteria | Satisfies 2 of the criteria | Satisfies 3 of the criteria | Satisfies all of the criteria | |
|  | · Layout of chairs enables accessible viewing of diagnostics |  |  |  |  | |
|  | · Layout of room allows accessible viewing of other team members |  |  |  |  | |
|  | · All members seated on a chair |  |  |  |  | |
|  | · Suitable venue in terms of location, temperature, lighting etc | (1) | (2) | (3) | (4) | |
| Technology & equipment | Availability of diagnostic equipment to view and share images and pathology with the team. | No radiology imaging facilities | Light box available with hard copy film | Current images available digitally with facilities for projecting/viewing images | Current images available digitally with facilities for projecting/viewing images and capability of accessing retrospective images (e.g. use of PACS) | |
|  | Availability of multiple screens scores extra 1 point. Score out of possible 9 is then standardised onto 1-4 scale to give overall rating. |  |  |  |  |  |
|  |  | (1) | (2) | (3) | (4) | |
|  |  | No histopathology facilities | Microscope | Microscope with facilities for projecting/viewing specimen/biopsy | Microscope with facilities for projecting and viewing specimen/biopsy and accessing retrospective data | |
|  |  | (1) | (2) | (3) | (4) | |
| **Meeting organisation and logistics** |  |  |  |  |  |  |
| Preparation prior to meetings: |  |  |  |  |  |  |
| a) agenda | Availability and content of agenda | No available agenda | Agenda, but limited info | | Comprehensive agenda | |
|  |  | (1) | (2) | | (3) | |
| b) prioritisation of complex cases | Prioritisation of complex cases on agenda to enable sufficient time for their discussion | No attempt is made to order cases in terms of complexity and an inappropriate amount of time is spent on cases (i.e. too much or too little) | Some attempt is made to order cases in terms of complexity but an inappropriate amount of time is spent discussing some of the cases | Patient cases are discussed in a clear order but time is used inappropriately in some cases | Patient cases are discussed in a clear order and an appropriate amount of time is spent discussing each case | |
|  |  | (1) | (2) | (3) | (4) | |
| Organisation/admin during meetings: |  |  |  |  |  |  |
| a) patient notes | Availability of patient notes | No patient records available at meeting | Some required past/current reports not available | Hardcopy and all necessary past/current reports available | Electronic access to patient notes and all necessary past/current reports available | |
|  |  | (1) | (2) | (3) | (4) | |
| b) case presentation | Comprehensiveness and coherence of case presentation | Rambling; entirely reading from notes; does not seem familiar with patient | Some evidence of familiarity with patient and info presented in reasonable fashion | | Comprehensive succinct coherent presentation (evidence of familiarity with patient and findings) | |
|  |  | (1) | (2) | | (3) | |
| **Clinical decision making** |  |  |  | |  | |
| Patient centred care | Includes mention of patient-based information (e.g. demography; co-morbidities; psycho-social or supportive needs; patient wishes/family preferences) | Patient-centred factors sufficiently acknowledged in less than 20% cases | Patient-centred factors sufficiently acknowledged in less than 50% cases | Patient-centred factors sufficiently acknowledged in 50% + cases (but not all cases) | Patient-centred factors sufficiently acknowledged in all cases | |
|  |  | (1) | (2) | (3) | (4) | |
| Treatment plans | Clarity of treatment plan | Treatment plan not discernible | Treatment plan communicated verbally | Treatment plan communicated verbally and recorded | Treatment plan communicated verbally, recorded with a clearly articulated plan regarding the next steps. | |
|  |  | (1) | (2) | (3) | (4) | |
| **Characteristic of Effective MDT-working** | **Quality Criteria** | **Quality of team-working (score in brackets)** | | | | |
| **Teamworking & culture** |  | **severe and sustained conflict** | **overt conflict un-sustained** | **tension sustained** | **tension un-sustained** | **no tension** |
|  |  | **(−4)** | **(−3)** | **(−2)** | **(−1)** | **(0)** |
| d) tension/conflict | Extent of tension and/or conflict observable in the team | ≥1clear example of conflict observed which persists throughout meeting | ≥1clear example of conflict observed does not persist throughout meeting | ≥1 instance of tension observed which persists throughout meeting | ≥1 instance of tension observed but does not persist throughout meeting | No tension |
