# Supplementary figures and images for: Nine keys for successful interprofessional collaboration Based on observing facilitators and barriers during different types of treatment meetings: A qualitative study
Source: PLoS One. 2026 Jul 1;21(7):e0350554. doi: 10.1371/journal.pone.0350554 (PMC13322501; doi:10.1371/journal.pone.0350554)

**Supplement 3 Figure. Final coding template.**

**
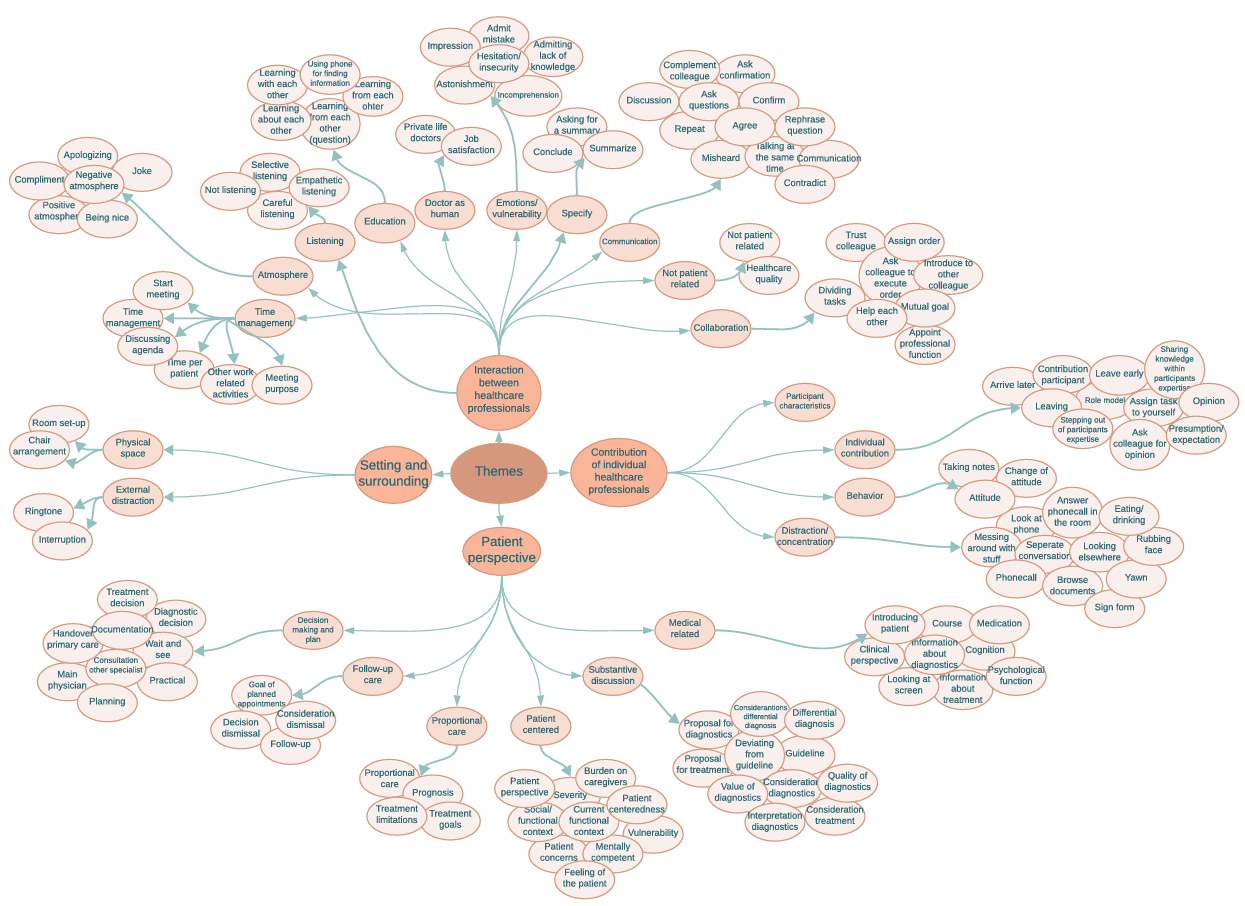
**

Supplement: S1 Fig — (DOCX) [file pone.0350554.s003.docx]
